# Comparative efficacy of peptide-based versus standard polymeric enteral nutrition in ICU patients at high nutritional risk: a multicenter randomized controlled trial

**DOI:** 10.1038/s41598-024-65277-w

**Published:** 2024-06-21

**Authors:** Rungsun Bhurayanontachai, Petch Wacharasint, Dujrath Somboonviboon, Chaiwut Sawawiboon

**Affiliations:** 1 Critical Care Medicine Unit, Department of Internal Medicine, Faculty of Medicine, Prince of Songkla University, Songkhla, Thailand; 2 Division of Pulmonary and Critical Care Medicine, Department of Internal Medicine, Phramongkutklao Hospital, Bangkok, Thailand; 3 Department of Emergency Medicine, Faculty of Medicine, Vajira Hospital, Navamindradhiraj University, Bangkok, Thailand

**Keywords:** Peptide-based formula, Enteral nutrition, Intensive care unit, Nutritional risk, Nutrition, Nutrition disorders, Respiratory tract diseases

## Abstract

Within intensive care units (ICU), the administration of peptide-based formulas (PBF) may confer nutritional advantages for critically ill patients identified with heightened nutritional risk. This investigation aimed to ascertain the efficacy of PBF in comparison to standard polymeric formulas (SPF) among this patient cohort. A double-blind, randomized controlled trial was conducted across three ICUs, encompassing 63 adult patients characterized by elevated modified Nutrition Risk in Critically Ill (mNUTRIC) scores. Enrollment occurred promptly subsequent to ICU admission, with participants allocated to receive either PBF or SPF. Primary outcome was the duration to achieve caloric targets. Secondary outcomes involved the evaluation of mean daily gastric residual volume, mechanical ventilation period, infection rates within the ICU, length of hospitalization, mortality rates, nutritional status and inflammatory markers, specifically serum albumin and interleukin-6 levels. Patients in the PBF group reached their caloric targets more expeditiously compared to the SPF group (2.06 ± 0.43 days versus 2.39 ± 0.79 days; p = 0.03). No significant differences were discernible between the groups regarding gastric residual volume, duration of mechanical ventilation, ICU length of stay, mortality, or infection rates. Both cohorts exhibited minimal adverse effects and were devoid of any instances of abdominal distension. While not reaching statistical significance, the observed trends in albumin and interleukin-6 levels suggest a potential advantage of PBF utilization. The implementation of PBF enabled swifter attainment of caloric goals in ICU patients at high nutritional risk without adversely impacting other clinical parameters. Given its favorable tolerance profile and potential immunomodulatory properties, PBF may be considered a valuable nutritional intervention in this setting.

Thai Clinical Trials Registry TCTR20220221006. Registered 21 February 2022, https://www.thaiclinicaltrials.org/show/TCTR20220221006.

## Introduction

Numerous critical care practice guidelines recommend the initiation of enteral feeding within 24–48 h of admission to the intensive care unit (ICU), which is known as early enteral nutrition (EN). This recommendation is based on the proven benefits of EN for physiological responses, enhanced clinical recovery, and a trend to reduce mortality rates^1–3^ when a standard polymeric formula (SPF) is continuously administered via a nasogastric feeding tube^4^. However, most critically ill patients experience feeding intolerance, including abdominal bloating, distension, aspiration, diarrhea, and large gastric residual volume (GRV)^5,6^. For patients who cannot tolerate SPF, instead of interrupting EN or acknowledging feeding failure, switching to a peptide-based formula (PBF) constitutes an alternative option for ensuring adequate nutritional support. Several clinical studies have shown the benefits of PBF over SPF, particularly for reducing the incidence and severity of undesired gastrointestinal (GI) symptoms, especially diarrhea, and improving gastric emptying^7–10^. However, the effects of PBF on other clinical outcomes remain controversial. Furthermore, the clinical studies that have been published thus far exhibit high heterogeneity in participants, diversity in feeding protocols, and the limited number of randomized double-blind studies^11–15^.

The recent guidelines from the American Society for Parenteral and Enteral Nutrition (ASPEN) recommend classifying nutritional risk using assessment tools such as Nutrition Risk Screening 2002 (NRS 2002), the Malnutrition Universal Screening Tool (MUST), the Nutrition Risk in the Critically Ill score (NUTRIC), or its modified version (mNUTRIC), to ascertain baseline nutritional status and to inform individualized nutritional management^4^. The mNUTRIC, widely used and validated across numerous clinical studies, correlates with clinical outcomes including mortality and adverse events in the intensive care unit^16–18^. In critically ill patients with an mNUTRIC score of ≥ 5, early caloric supplementation has been linked to reduced mortality and morbidity by providing higher energy and protein, and by facilitating the rapid achievement of caloric goals^19–22^. However, some studies have reported findings that contradict these benefits^23–25^. Consequently, it remains uncertain whether nutritional risk-based management, encompassing calorie and protein intake as well as formula composition, should be considered when implementing nutritional supplementation in critically ill patients, particularly those at high nutritional risk.

Consequently, within a cohort of critically ill patients characterized by substantial nutritional risk as quantified by the mNUTRIC score, this study sought to elucidate the comparative effects of PBF versus SPF. The clinical endpoints evaluated included the time required to achieve caloric goals, mean daily gastric residual volume (GRV), duration of mechanical ventilation in days, incidence of ICU-acquired infections, length of ICU stay, ICU mortality, and the variation in biochemical indicators reflective of inflammatory and nutritional status.

## Materials and methods

### Study design

This prospective, randomized, double-blind, multicenter trial was conducted in the ICUs and respiratory care units (RCU) at Songklanagarind Hospital, Songkhla; Phramongkutklao Hospital Bangkok; and Vajira Hospital, Bangkok between January 2019 and June 2022.

### Study populations

All mechanically ventilated patients who were admitted to the ICU or RCU during the study period (January 2019 to June 2022) were screened based on the inclusion criteria: (1) age ≥ 18 years; (2) body mass index (BMI) 18–30 kg/m^2^, (3) anticipated mechanical ventilation for ≥ 2 days; (4) risk of malnutrition, indicating a requirement for nutritional support [based on mNUTRIC Score ≥ 5 points]; (5) hemodynamically stable and indicated to receive enteral feeding via nasogastric tube within the first 48 h of ICU admission; and (6) anticipated exclusive nasogastric feeding duration ≥ 5 days.

We excluded patients: (1) with a risk or diagnosis of aspiration pneumonia; (2) with thyroid disorders; (3) with severe hepatic impairment (Child–Pugh Score, Class C); (4) on renal replacement therapy; (5) with suspected or diagnosed abdominal hypertension; (6) with HIV infection or autoimmune diseases; (7) with uncontrolled cancer or terminal disease; (8) who received chemotherapy or radiotherapy in the preceding 6 months; (9) continuously received any immunosuppressants for ≥ 2 weeks within the 4 months prior to ICU admission; (10) documented history of hypersensitivity to any ingredients of the feeding formula; (11) patients requiring additional calorie and nutrient supplementation, besides EN through nasogastric feeding, to achieve caloric goals during the trial (e.g. parenteral nutrition). Furthermore, patients who required fentanyl > 2 μg/kg/h, morphine > 0.05 mg/kg/h, or norepinephrine > 0.3 μg/kg/min were excluded.

### Ethical approval

The trial protocol was developed in accordance with the ethical principles outlined in the Declaration of Helsinki and ICH Good Clinical Practice guideline for medical research involving human subjects. The trial protocol and informed consent form was approved by all participating institutes, including the Human Research Ethics Committee (HREC) Faculty of Medicine, Prince of Songkla University (REC.61–124-14–1), the Institutional Review Board (IRB) of the Royal Thai Army Medical Department (IRB no. P003h/61), and the Research Ethics Committee of the Faculty of Medicine Vajira Hospital, Navamindradhiraj University (IRB no. 036/61).

This study was registered in the Thai Clinical Trials Registry (reg. no. TCTR20220221006). All participants or their legally authorized representatives provided written informed consent before enrollment in the study.

### Study protocol

Block randomization was conducted via a computerized system sponsored by the funding entity, with stratification by study site location. Participants were assigned in equal numbers to either the PBF or the SPF. Subsequent to patient recruitment and randomization, each study site accessed the randomization sequence through a secured telephone system. The trial was designed to be double-blind; as such, all involved parties—including treating clinicians, caregivers, participants, researchers, and the sponsor—were kept unaware of the specific enteral formulas administered throughout the study period. The formulas were provided as powders labeled 'Product A' or 'Product B', with packaging that was intentionally made indistinguishable to preserve the integrity of the blind. These powders were then prepared with a caloric density of 1 kcal/mL by hospital dietitians or nurses. The comprehensive biochemical compositions of PBF and SPF are presented in Table 1.

**Table 1:** The study formula at the specific concentration of 1 kcal/1 mL.

	Peptide-based formula (PBF)	Standard polymeric formula (SPF)
% Caloric distribution (Protein: Carbohydrate: Fat)	20:45:35	20:52:28
Source of protein	Whey protein hydrolysate Leucine	Sodium caseinate Soy protein isolate
Source of carbohydrate	Maltodextrin Potato starch	Maltodextrin Sucrose Fructo-oligosaccharide
Source of fat	Fish oil Canola oil MCT oil	Rice bran oil MCT oil
Osmolarity (mOsm/L)	242	307
Osmolality (mOsm/kg H_2_O)	290	364

Based on the estimated or actual body weight, the target energy requirement for the first 3 days post-randomization was set at 25 kcal/kg/day, which was then increased to 30 kcal/kg/day from the fourth day until the 14th day or the end of the study period. No alternative routes of nutritional support or dietary supplementation for participants were permitted during the trial.

### Enteral feeding protocol

Enteral nutrition (EN) was administered via a continuous infusion starting at an initial rate of 20 mL/hour, employing an infusion pump to deliver the enteral formula through a nasogastric tube. Gastric residual volumes (GRVs) were manually aspirated every six hours to monitor for signs of feeding intolerance. In instances where the GRV was below 250 mL, the infusion rate was escalated by 20 mL/hour at each six-hour interval until the prescribed caloric intake was achieved. Conversely, for patients exhibiting elevated GRVs exceeding 250 mL, the infusion rate was either halved or maintained at the minimal rate of 20 mL/hour, in conjunction with the intravenous administration of metoclopramide at a dose of 10 mg every six to eight hours. This regimen was continued until GRV levels were reduced to acceptable thresholds, upon which the rate of EN was progressively increased towards the target rate. It is noteworthy that, owing to the lack of available intravenous erythromycin within the country, intravenous metoclopramide constituted the conventional therapeutic approach for managing elevated GRVs in critically ill patients demonstrating feeding intolerance. This treatment protocol was maintained for the duration of the study.

### Data collection

At enrollment, demographic data, including age, sex, underlying medical conditions, reasons for admission, body weight, and height, were recorded. Biochemical parameters, including blood urea nitrogen (BUN) and creatinine (Cr), liver function tests, and blood electrolyte levels, along with a complete blood count (CBC) and coagulogram, were performed. Using biochemical tests, nutritional status, including measurements of prealbumin, retinol-binding protein, albumin, and 24-h urinary nitrogen, was assessed. Moreover, the levels of inflammatory biomarkers, such as C-reactive protein (CRP), interleukin-6 (IL-6), and procalcitonin, were determined. Illness severity was evaluated at ICU admission using the Acute Physiology and Chronic Health Evaluation II (APACHE II) and Simplified Organ Failure Assessment (SOFA) scores.

The Bhumibol Adulyadej Hospital Nutrition Triage (BNT) was employed for the subjective assessment of the patients' nutritional status. The BNT incorporates aspects such as caloric intake history; unintentional weight loss; edema signs; subcutaneous fat and muscle loss examination; functional status; and the presence of acute, subacute, or chronic diseases that may cause inflammatory processes or metabolic derangements and is a validated tool for the nutritional evaluation of Thai adult patients. Based on the overall BNT score, nutritional status can be categorized into four strata: NT-1 (no risk of malnutrition; 0–4 points), NT-2 (mild malnutrition; 5–7 points), NT-3 (moderate malnutrition; 8–10 points), and NT-4 (severe malnutrition; > 10 points)^26^.

Clinical and BNT evaluations were performed daily. The total daily caloric and protein supplementation, as well as total and average daily GRV, were recorded. Biochemical parameters, including CBC, serum electrolytes, liver, and renal function tests (BUN and Cr), retinol-binding protein, prealbumin, albumin, CRP, procalcitonin, and IL-6 levels, were analyzed at baseline and every other day for 14 days.

The 24-h urinary collection for nitrogen and creatinine measurements was undertaken on days 1, 3, 5, and 14. The nitrogen balance was calculated using a standard formula described previously^27^.

### Safety profile

All signs and symptoms of GI intolerance, such as vomiting, diarrhea, constipation, abdominal distention, and aspiration, were daily monitored and documented by researchers as adverse events (AE). Diarrhea assessment employed the Hart and Dobb score based on stool consistency and volume, wherein a score ≥ 12 indicates diarrhea^28^.

The incidence of diarrhea was calculated by dividing the number of days that the patient had a Hart and Dobb score ≥ 12 by the patient’s total duration (days) of study participation. All AEs were recorded in an AE report form, and the researchers adjusted the relationship between these events and the study formula. AEs were continuously monitored and followed-up during the study and for 28 days after study completion. The institutional ethics committee was notified of these records per standard regulations.

### Study outcomes

The primary endpoint of this study was the duration required to meet the established caloric goals between both enteral formulations. Secondary predefined endpoints encompassed the mean daily GRV, the rate and total dose of administration of intravenous metoclopramide as a prokinetic therapy, the number of days on mechanical ventilation, the incidence rate of infections acquired in the ICU, the total duration of ICU stay, the mortality rate within the ICU, and the fluctuations in serum markers indicative of inflammation and nutritional status. Additionally, the study monitored the frequency of adverse events (AEs) and the occurrence of diarrhea within the respective groups.

### Statistical analysis

In a prior retrospective study of patients with acute gastrointestinal injury, a significant difference of 31 h was observed in the time to achieve caloric goals between the PBF and SPF groups^29^ This discrepancy guided our calculation of sample size, which was designed to accommodate a type I error (alpha) of 0.1 and a type II error (beta) of 0.2, yielding a requirement of 39 participants for each group.

Continuous variables were presented as the mean and standard deviation (SD), whereas categorical variables were expressed as the frequency and proportion. Intergroup differences in patient characteristics and outcomes of interest were assessed using the student’s *t*-test, Wilcoxon rank-sum test, Fisher's exact test, and chi-square test, as appropriate.

For repeated-measure analysis, intergroup comparison of variables was undertaken using generalized estimating equations (GEE) with a population-average model. The working correlation matrix for GEE analysis was selected based on the lowest quasi-likelihood under the independent model criterion (QIC). Interactions between the group and time were quantified as β-coefficients with 95% Wald confidence intervals (CI).

The proportion of patients achieving the caloric goal was depicted using a Kaplan–Meier curve and compared between the groups via the Logrank test. Concerning nutritional status, which was determined daily by the BNT score, we categorized the BNT score into four strata as previously described. We then conducted pairwise comparisons within the groups for three-time intervals: between day 1 and day 7, day 1 and day 14, and day 7 and day 14. These comparisons aimed to determine changes in BNT score strata using the chi-square test. Additionally, pairwise comparisons of BNT score classification on days 1, 7, and 14 were analyzed using the chi-square test to assess differences between the groups.

Data were analyzed on an intention-to-treat basis. No imputation was made for missing data; *P* < 0.05 was considered statistically significant. Data analyses were performed using the IBM SPSS Statistics for Macintosh (version 27.0; IBM Corp., Armonk, NY), and MedCalc® Statistical Software version 22.017 (MedCalc Software Ltd, Ostend, Belgium; https://www.medcalc.org; 2024).

## Results

### Baseline clinical characteristics

**Figure 1:**
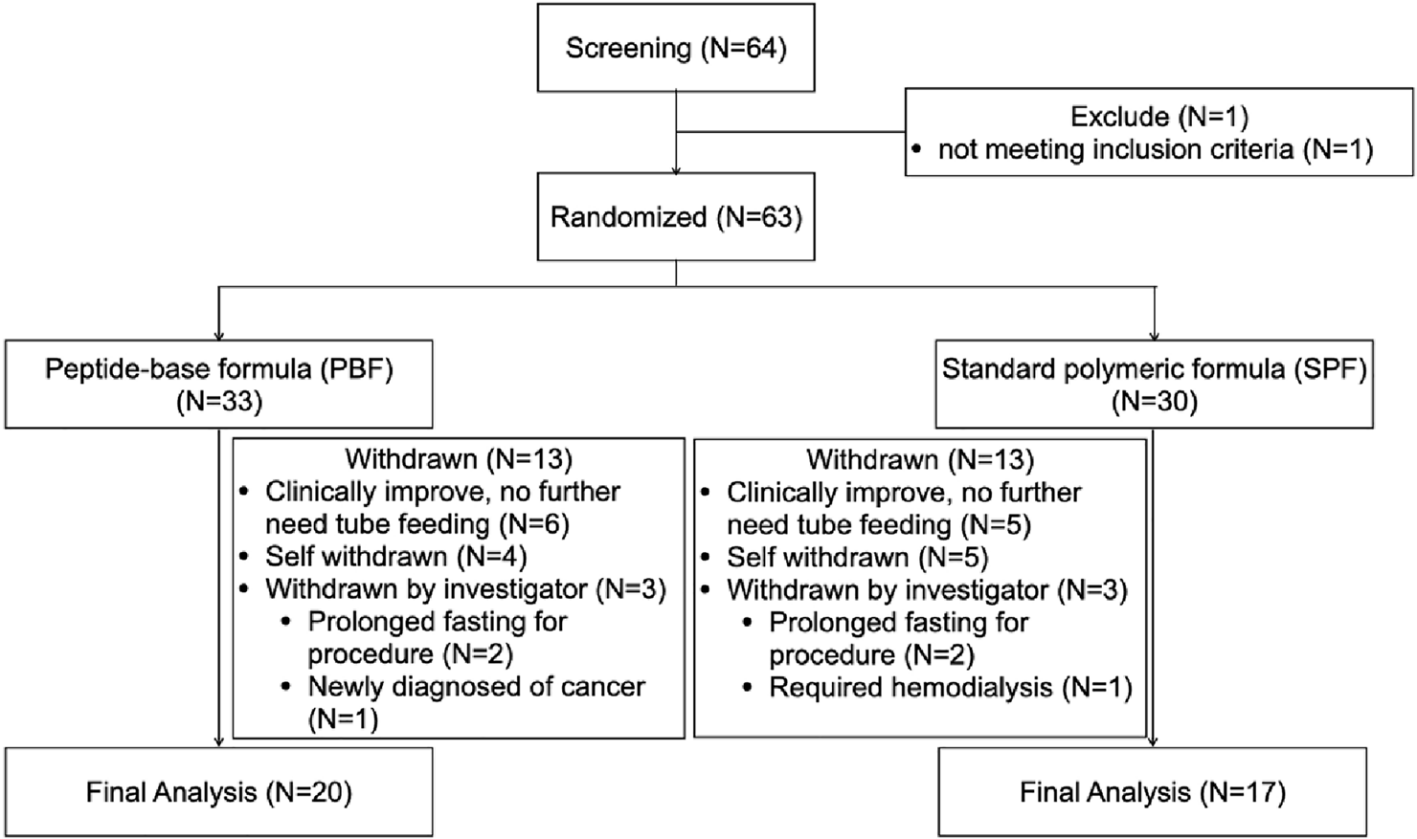
Patients flow diagram.

During the study period, from among 64 patients who were assessed; one was excluded because of an mNUTRIC score of less than 5. Consequently, 63 patients were randomized to the PBF (n = 33) and SPF (n = 30) groups (Fig. 1). Comparison of demographic data, as illustrated in Table 2, revealed no significant intergroup difference in mean age, sex distribution, body mass index (BMI), Glasgow Coma Scale scores, APACHE II scores, SOFA scores, mean arterial blood pressure, or use of vasopressors or opioid analgesics.

**Table 2:** Intergroup comparison of demographic and clinical characteristics.

	Peptide-based formula (PBF) (n = 33)	Standard polymeric formula (SPF) (n = 30)	p
Demographic data			
Age (year)	71.39 ± 14.96	72.90 ± 14.91	0.69
Female: N (%) *	17 (51.5%)	15 (50.0%)	0.91
BMI (kg/m^2^)	21.48 ± 2.75	22.21 ± 2.45	0.27
Glasgow Coma Scale	8.58 ± 3.46	8.50 ± 3.47	0.93
APACHE II Score	27.39 ± 7.98	27.13 ± 5.34	0.88
SOFA Score	7.12 ± 3.31	7.07 ± 2.70	0.94
Mean arterial blood pressure (mmHg)	94.41 ± 24.50	98.36 ± 22.34	0.51
Vasopressor use, N (%)	13 (39.4%)	9 (30.0%)	0.44
Opioid analgesia use, N (%)	20 (60.6%)	15 (50.0%)	0.40
Cause of ICU admission, N (%) • Respiratory disease • Cardiovascular disease • Neurological disease • Sepsis	4 (12.1%) 4 (12.1%) 14 (42.4%) 11 (33.3%)	5 (16.7%) 7 (23.3%) 15 (50.0%) 3 (10.0%)	0.14
mNUTRIC Score	6.27 ± 1.18	6.57 ± 1.14	0.32
BNT score	8.39 ± 2.76	6.83 ± 2.69	0.03
BNT by category, N (%)* • NT-1 • NT-2 • NT-3 • NT-4	3 (9.1%) 8 (24.2%) 14 (42.4%) 8 (24.2%)	5 (16.7%) 15 (50.0%) 6 (20.0%) 4 (13.3%)	0.07
Laboratory tests			
Hemoglobin (g/dL)	10.07 ± 2.06	10.55 ± 2.00	0.35
White blood cell count (× 10^3^/µL)	13.17 ± 5.29	13.49 ± 6.66	0.83
Total lymphocytes count (cell/µL)	1,093.11 ± 1,016.75	1,176.77 ± 1,052.79	0.75
Blood urea nitrogen (mg/dL)	28.28 ± 16.05	36.45 ± 23.82	0.12
Creatinine (mg/dL)	1.09 ± 0.68	1.42 ± 1.19	0.18
Total bilirubin (mg/dL)	1.19 ± 1.80	1.19 ± 1.30	0.99
Magnesium (mg/dL)	1.87 ± 0.34	2.06 ± 0.32	0.03
Phosphorus (mg/dL)	2.72 ± 0.88	2.99 ± 1.32	0.35
Lactate (mmol/L)	14.74 ± 10.00	47.55 ± 179.93	0.30
Nutritional biochemical markers			
Albumin (g/dL)	2.73 ± 0.72	2.94 ± 0.63	0.22
Prealbumin (mg/dL)	9.12 ± 5.78	11.76 ± 5.31	0.07
Retinol binding protein (mg/dL)	3.06 ± 2.79	3.15 ± 2.47	0.90
Inflammatory markers			
C-reactive protein (mg/L)	159.78 ± 94.96	113.32 ± 88.02	0.05
Procalcitonin (ng/mL)	11.67 ± 20.01	6.68 ± 14.38	0.26
Interleukin-6 (pg/mL)	325.30 ± 647.11	84.87 ± 112.60	0.06

Data are presented as the mean ± SD and were analyzed by the student’s *t*-test.

*Data are presented as the frequency and proportion [n (%)] and were analyzed by the chi-square test.

The causes of ICU admission were classified as respiratory, cardiovascular, and neurological diseases and sepsis, without significant disparity in the distribution across groups. However, nutritional risk, assessed using the mNUTRIC and BNT scores, differed significantly, with the PBF group exhibiting a higher BNT score (8.39 ± 2.76 versus 6.83 ± 2.69; p = 0.03).

Laboratory tests, including hemoglobin levels, white blood cell count, total lymphocyte count, BUN, Cr, total bilirubin, and phosphorus levels, showed no significant intergroup differences. Nonetheless, magnesium levels were notably higher in the SPF group (1.87 ± 0.34 mg/dL vs. 2.06 ± 0.32 mg/dL; p = 0.03).

Nutritional biochemical markers, such as albumin, prealbumin, and retinol-binding protein levels, did not differ significantly between the groups. Inflammatory markers, including CRP, showed a higher trend in the PBF group, and interleukin-6 levels were also elevated in this group, although the difference was not statistically significant.

### Primary and secondary outcomes

After randomization, a total of 26 patients, 13 from each group, withdrew from the study. Consequently, 20 patients in the PBF group and 17 in the SPF group completed the 14-day study period, as depicted in Fig. 1. The analysis of outcomes was performed on an intention-to-treat basis, the results of which are presented in Tables 3 and 4.

**Table 3:** Outcomes.

	PBF (n = 33)	SPF (n = 30)	p
Primary outcomes			
Time to reach the caloric goal (25 kcal/kg/day) (days) *	2.06 ± 0.43	2.39 ± 0.79	0.03
Secondary outcomes			
Daily Average GRV (mL) *			
• Day 1	51.65 ± 75.42	29.60 ± 68.23	0.23
• Day 2	42.54 ± 59.57	46.22 ± 89.80	0.85
• Day 3	46.35 ± 58.72	30.35 ± 78.43	0.37
• Day 4	28.32 ± 52.73	31.76 ± 75.41	0.84
• Day 5	19.84 ± 50.96	11.70 ± 19.51	0.46
• Day 6	31.56 ± 68.94	9.24 ± 12.22	0.12
• Day 7	31.49 ± 67.93	12.79 ± 24.17	0.22
• Day 8	21.25 ± 43.77	11.83 ± 24.13	0.40
• Day 9	22.71 ± 49.70	14.05 ± 23.10	0.49
• Day 10	30.65 ± 58.47	11.22 ± 17.98	0.17
• Day 11	26.01 ± 45.93	9.63 ± 17.88	0.15
• Day 12	24.63 ± 57.32	8.79 ± 15.26	0.26
• Day 13	16.04 ± 25.02	2.87 ± 5.32	0.04
• Day 14	19.00 ± 44.27	3.83 ± 7.58	0.19
Metoclopramide used, N (%) **	15 (45.5%)	8 (26.7%)	0.12
Total metoclopramide dose, (mg) *	59.55 ± 123.39	31.33 ± 67.61	0.27
ICU mortality, N (%) **	1 (3.0%)	3 (10.0%)	0.26
ICU length of stay, (days) *	9.27 ± 4.54	8.50 ± 4.00	0.48
Duration of mechanical ventilation, (days) *	7.88 ± 5.09	7.10 ± 4.24	0.51
Nosocomial/ ICU infection, N (%) **	3 (9.1%)	3 (10.0%)	0.90
Adverse events, N (%) **			
Diarrhea	3 (9.1%)	3 (10.0%)	0.90
Vomiting	1 (3.0%)	2 (6.7%)	0.50
Constipation	5 (15.2%)	3 (10.0%)	0.54
Abdominal distension	0 (0%)	0 (0%)	NA

*Data are presented as the mean ± SD and were analyzed by the student’s *t*-test.

**Data are presented as the frequency with proportion [ N (%)] and were analyzed by using the chi-square test.

**Table 4:** Comparison of repeated measurement variables by GEE, compared to SPF.

Parameters	β-coefficient	Standard Error	95% Wald CI	p
Daily average GRV, mL	0.16	1.51	− 2.44 to 3.48	0.73
BNT, point	− 0.17	0.11	− 0.39 to 0.04	0.12
Retinol binding protein, mg/L	0.07	0.16	− 0.24 to 0.38	0.66
Prealbumin, mg/dL	− 0.41	0.40	− 1.20 to 0.38	0.31
Total lymphocyte count, cell/μL	− 13.15	40.90	− 93.31 to 67.01	0.75
Albumin, g/dL	0.31	0.16	− 0.001 to 0.63	0.05
Nitrogen balance, g/day	− 0.08	0.13	− 0.34 to 0.19	0.56
C-reactive protein, mg/L	− 4.99	4.87	− 14.54 to 4.56	0.31
Interleukin-6, pg/mL	− 34.31	18.11	− 69.81 to 1.19	0.06
Procalcitonin, ng/mL	− 0.65	0.70	− 2.02 to 0.72	0.35
Hart–Dobb diarrhea score	0.17	0.10	− 0.02 to 0.37	0.08

**Figure 2:**
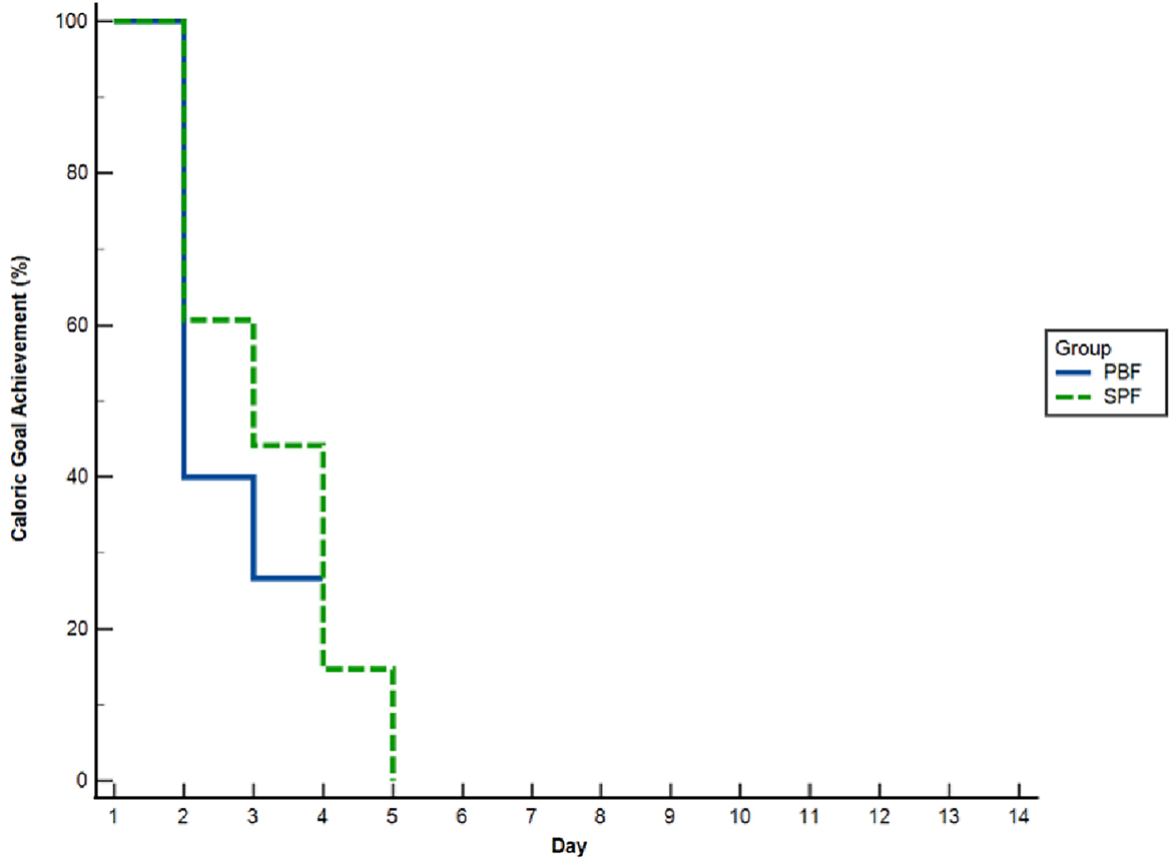
Kaplan–Meier curve depicting the time to achieve caloric goals in patients receiving Peptide-based (PBF) versus Standard Polymeric Formula (SPF) nutrition. In the 14-day study, PBF participants (solid blue line) and SPF participants (dashed green line) showed divergent caloric intake patterns, as depicted in Kaplan–Meier curves. The PBF group had a less pronounced decline in achieving caloric goals compared to SPF. However, Logrank test analysis indicated no significant difference (p = 0.21) in caloric goal attainment between the groups over the duration of the study.

The primary outcome demonstrated a statistically significant reduction in the time to achieve the caloric goal (25 kcal/kg/day) for the PBF group, taking 2.06 ± 0.43 days, compared to 2.39 ± 0.79 days for the SPF group (p = 0.03). The percentage of patients reaching their caloric goals was illustrated by a Kaplan–Meier curve (refer to Fig. 2), which shows the PBF group achieving caloric goals more consistently over the 14-day period than the SPF group. Nonetheless, despite the variance in patterns of achievement, the Logrank test yielded a p-value of 0.21, indicating no statistically significant difference in caloric goal achievement between the PBF and SPF groups during the study.

No statistically significant difference was observed in the daily average GRV, as indicated by a β-coefficient of 0.16 (95% CI − 2.44 to 3.48, p = 0.73). Furthermore, no significant differences were found between the groups for secondary outcomes, including metoclopramide use (45.5% in the PBF group vs. 26.7% in the SPF group, p = 0.12) and ICU mortality rates (3.0% in PBF vs. 10.0% in SPF, p = 0.26). The duration of ICU stays, length of mechanical ventilation, and incidence of nosocomial infections were comparable for both PBF and SPF groups.

For the BNT score, the β-coefficient was − 0.17 (95% CI: − 0.39 to 0.04, p = 0.12), indicating no statistically significant difference between groups. Similarly, measurements of retinol-binding protein, prealbumin, total lymphocyte count, and nitrogen balance revealed no significant intergroup differences. However, albumin levels showed a β-coefficient of 0.31 (95% CI: − 0.001 to 0.63, p = 0.05), suggesting a potential trend towards statistical significance. Furthermore, IL-6 levels exhibited a β-coefficient of − 34.31 (95% CI: − 69.81 to 1.19, p = 0.06), approaching significance. The Hart-Dobb diarrhea score also indicated a non-significant trend toward significance, with a β-coefficient of 0.17 (95% CI: − 0.02 to 0.37, p = 0.08) as detailed in Table 4.

**Figure 3:**
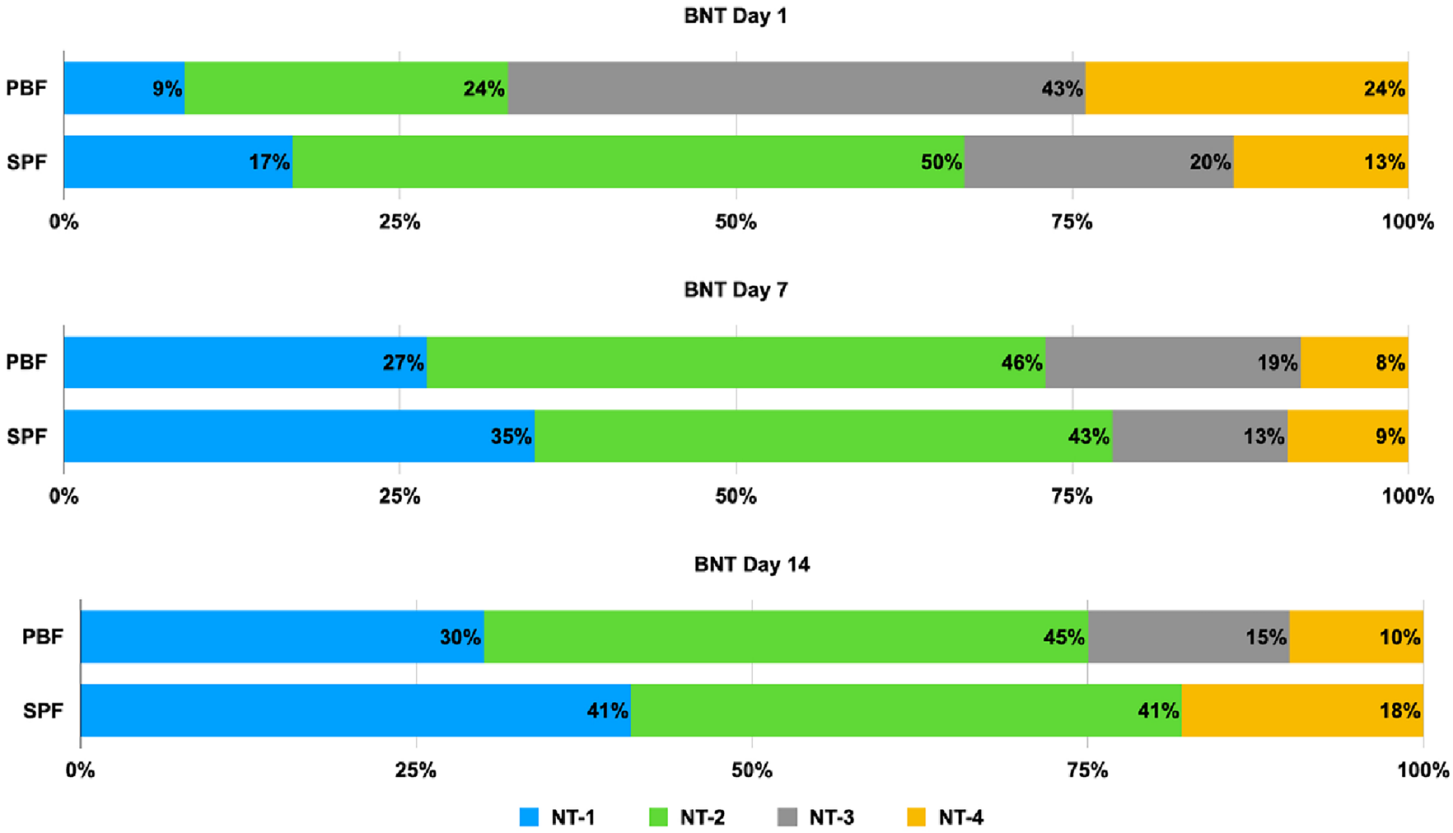
Stacked bar chart illustrating the distribution of BNT score classifications on days 1, 7, and 14 for patients receiving Peptide-based Formula (PBF) compared to those on Standard Polymeric Formula (SPF). Pairwise comparisons of the percentage of patients in each BNT score classification between PBF and SPF on days 1, 7, and 14 showed no significant differences (p = 0.07, 0.9, and 0.35, respectively). However, significant changes were observed in the proportion of patients within each BNT stratum from day 1 to day 7 (p = 0.02), and from day 7 to day 14 (p < 0.01) in the PBF group. Similarly, changes in BNT classification in the SPF group followed the same pattern, with significant differences noted (p < 0.01 and p < 0.01, respectively).

Significant alterations in BNT classification within the PBF and SPF groups were noted between days 1 and 7, and again between days 7 and 14, as illustrated in Fig. 3. During the study, the PBF group predominantly maintained a higher percentage of NT-3, while NT-2 was the prevalent classification in the SPF group. For both groups, the proportion of NT-1 and NT-2 increased from Day 1 to Day 14, whereas the proportion of NT-3 and NT-4 decreased. Notably, the SPF group showed a consistently greater percentage of NT-1 and NT-2, particularly on Days 7 and 14. Over time, the range of BNT classifications in the SPF group narrowed, with NT-2 becoming increasingly dominant by Day 14. Despite these trends, pairwise comparisons between the groups on days 1, 7, and 14 did not yield statistically significant differences, as indicated by p-values of 0.07, 0.9, and 0.35, respectively.

### Adverse events

Throughout the study, there were 15 reported adverse events (AEs), predominantly mild in nature. The frequency of AEs, such as diarrhea, vomiting, and constipation, was not significantly different between the PBF and SPF groups (p = 0.90, 0.50, and 0.54, respectively). Neither abdominal distension nor any major AEs were reported in either group.

## Discussion

The early implementation of EN in intensive care units is empirically validated to improve physiological responses, expedite patient recovery, and decrease mortality rates^1–3^. The preference for PBF over SPF among patients with feeding intolerance is underpinned by its benefits in alleviating gastrointestinal symptoms and enhancing gastric emptying^9^. However, claims of PBF's superior clinical outcomes remain a subject of discussion, due in part to the diversity of patient demographics and the varied feeding protocols across studies^7,12,14,30^.

Our study—a randomized, controlled, double-blind, multicenter trial—was meticulously structured to reduce variability and facilitate a robust comparison between PBF and SPF in terms of achieving caloric targets, managing daily average GRV, influencing ventilator dependency, ICU infection rates, the duration of ICU stay, ICU mortality, and specific biochemical markers. This was particularly focused on critically ill patients identified as having a high nutritional risk, as determined by a substantial mNUTRIC score. While our results are in agreement with prior studies, demonstrating a notable reduction in the time to achieve caloric goals with PBF, we did not observe a parallel decline in daily average GRV, mechanical ventilation requirements, ICU stay length, or mortality rates. These findings suggest that while PBF may accelerate the achievement of nutritional targets, this does not inherently equate to wider clinical improvements.

In PBF, the application of enzymatic hydrolysis to whey protein facilitates the generation of diminutive peptide constituents. Notably, dipeptides and tripeptides are expedited in their absorption by enterocytes, mediated through peptide transporters such as PepT1, with this process being particularly accentuated during instances of gastrointestinal inflammation, attributable to direct or collateral injury in patients experiencing critical illnesses. Furthermore, the pronounced incorporation of medium-chain triglycerides (MCT) within these PBF, in stark contrast to the long-chain triglycerides (LCT) that are a hallmark of SPF, significantly augments the mucosal absorption of nutrients. This enhancement is of paramount importance for patients in critical care settings who are afflicted with intestinal failure and concomitant gut mucosal injury, thereby potentially ameliorating their nutritional status and clinical outcomes^9^.

The findings of this study align with those of recent research, such as that conducted by Wang et al.^10^, who explored the effects of small peptide formulas on critically ill patients, particularly those with acute GI injury. Although no mortality differences were reported, enhanced protein intake and reductions in ICU and hospital stay durations were noted. Nguyen et al.^31^, found that PBF with 100% whey protein facilitated lower gastric residual volumes and enhanced glycemic control in critically ill adults. Similarly, Ibrahim et al.^8^ observed that critically ill pediatric patients receiving PBF met their caloric goals more rapidly and exhibited better GI tolerance than those receiving SPF. Our 14-day study aimed to evaluate the long-term effects of various nutritional parameters; however, the benefits of PBF were predominantly evident in the short term. This was demonstrated by a swifter achievement of caloric goals and an early separation in the Kaplan–Meier curves, which illustrated the rate of caloric goal attainment between groups. We hypothesize that the favorable outcomes linked to PBF may stem from the biochemical characteristics of small peptides, notably their expedited enterocyte absorption and potential immunomodulatory impact^31^.

In contrast to our findings, Cateron et al.^11^ observed no significant differences in GI tolerance or daily energy intake between semi-elemental and polymeric formulas in critically ill patients with brain injury. This suggests that specific injuries and conditions may mediate the response to different types of EN. Although our study identified variations in caloric composition and macronutrient sources between PBF and SPF, it is important to note that the observed benefits of PBF, such as the accelerated achievement of caloric goals, may not be solely ascribed to peptide content.

PBF have been associated with immunomodulatory effects in various inflammatory diseases. In the critical care setting, patients often experience complex inflammatory responses, such as those triggered by infections and trauma, which serve as defense mechanisms against physiological stress. Consequently, PBF may have the capacity to attenuate these inflammatory responses and modulate the inflammatory cascade, potentially preserving organ function during periods of critical illness^32^. Although the biochemical markers assessed in our study—including both inflammatory and nutritional indicators—generally showed no significant differences, the trends observed in albumin and interleukin-6 levels hinted at a potential influence of PBF on specific nutritional and inflammatory parameters. The absence of statistically significant findings in our cohort suggests a need for further investigation, potentially through more extensive or longer-term studies, to elucidate the effects of PBF.

Rattanachaiwong et al.^12^ reported a reduction in stool weight and diarrhea frequency when using PBF, a finding that is in line with our results, which showed no significant difference in the incidence of diarrhea or Hart-Dobb scores between PBF and SPF groups. Such outcomes lend further support to the safety profile of PBF, corroborating our findings that PBF is as safe as SPF for use in critically ill adult patients.

Although the current consensus suggests the use of SPF as the first-line regimen for every critically ill patient^4^, we advise the initial use of PBF for critically ill patients presenting with high nutritional risk or GI dysfunction, including malabsorption or dysmotility issues. In such clinical scenarios, PBF may alleviate feeding intolerance, facilitate the attainment of caloric objectives, and reduce gastrointestinal side effects. Although current guidelines advocate for the use of indirect calorimetry (IC) to ascertain energy expenditure in mechanically ventilated, critically ill patients^4,33^, it is important to recognize that this technology is not universally available in our institutions. Consequently, our standard ICU practice relies on a predictive equation, starting with 25 kcal/kg/day for the initial three days and increasing to 30 kcal/kg/day after a thorough assessment. Additionally, a post-hoc analysis of the PermiT trial indicated no significant difference in 90-day mortality between hypocaloric and standard feeding regimes, irrespective of the patients' nutritional risk^24^. Therefore, the caloric management approach adopted in our study is unlikely to have affected the primary and secondary outcomes within our cohort.

Our study's methodological rigor is fortified by stringent adherence to well-defined inclusion and exclusion criteria and the employment of the mNUTRIC score for nutritional risk assessment, which collectively ensured a homogeneous study population and accurate nutritional evaluation. Furthermore, we utilized the Generalized Estimating Equations approach, a sophisticated statistical analysis for repeated measures. This technique was chosen to mitigate bias associated with time-variant factors and unobservable heterogeneity within and between subjects, offering an advantage over traditional methods for repeated measurements^34^.

Despite yielding some significant results, our study faced limitations, particularly the small sample size that did not meet the calculated requirement and a high dropout rate in both groups, which may have compromised our ability to detect meaningful differences in the primary and secondary outcomes. The strict inclusion criteria, combined with the recruitment challenges during the pandemic, notably slowed patient enrollment. Budgetary constraints further necessitated an early halt to enrollment, thus hindering the achievement of the desired sample size. Moreover, the focus on a medical ICU population primarily comprising patients with neurological conditions and sepsis calls for a cautious interpretation of the results due to the complexity of critical illnesses. While we observed a statistically significant reduction in time to reach caloric goals in the PBF group—approximately 0.33 days or 8 h—the clinical significance of this finding in practical settings may be limited. Additionally, the absence of statistically significant differences in the secondary outcomes suggests that the quicker caloric attainment with PBF may not confer broader clinical advantages. Therefore, more comprehensive clinical trials are essential to affirm the benefits of PBF over SPF in critical care.

## Conclusion

In conclusion, our study corroborates existing research, highlighting PBF's potential in enhancing feeding tolerance and meeting nutritional targets for critically ill patients. Yet, given the diversity of patient profiles, small sample size, clinical settings, and outcome measures, interpretations must be made cautiously. Notwithstanding the positive implications for PBF in critical care nutrition, our results call for further research to thoroughly understand PBF's benefits and to evaluate its effectiveness across different critical care conditions.

## Data Availability

The data that support the findings of this study are available on request from the corresponding author, RB.

## Abbreviations

APACHE II: Acute physiology and chronic health evaluation II
BNT: Bhumibol adulyadej hospital nutrition triage
EN: Enteral nutrition
GEE: Generalized estimating equations
GRV: Gastric residual volume
ICU: Intensive care unit
mNUTRIC: Modified nutrition risk in critically Ill
PBF: Peptide-based formula
RCU: Respiratory care unit
SOFA: Simplified organ failure assessment
SPF: Standard polymeric formula
